# Is the Grass Always Greener in Suburban Neighborhoods? Outdoors Play in Suburban and Inner-City Neighborhoods

**DOI:** 10.3390/ijerph14070759

**Published:** 2017-07-11

**Authors:** Mika R. Moran, Pnina Plaut, Dafna Merom

**Affiliations:** 1 Faculty of Architecture and Town Planning, Technion—Israel Institute of Technology, Haifa 320003, Israel; pninatech@gmail.com; 2 School of Science and Health, Western Sydney University, Penrith, NSW 2751, Australia; D.Merom@westernsydney.edu.au

**Keywords:** outdoors play, neighborhood, mixed methods, children, physical activity

## Abstract

Children’s outdoors play (OP) is an important source of physical activity that has been decreasing in recent years due to changes in neighborhood design, parent safety concerns and child sedentary leisure. However, few studies examined such determinants from children’s perspectives. This study explores environmental and socio-cultural aspects of children’s OP using a qualitative and quantitative approach. Data was collected in two phases: (1) a survey on OP and related variables among 5th and 6th graders (10–12 years old) (*n* = 573); and (2) a mapping activity and semi-structured interview among a subsample of the survey (*n* = 80). The most common locations for routine OP were parks (40%) followed by public facilities (26%) and streets (17%). OP was significantly associated with perceived environment, independent mobility and gender, but not with neighborhood type. Inner-city participants reported a higher number and greater variety of OP areas (23 vs. 14). Three main barriers of OP were identified—low quality and poorly maintained play areas, other people in public spaces, and social norms that undermine OP. Thus, in order to encourage routine OP, environmental change to create safe and attractive OP settings should be accompanied by community interventions to enhance social norms that are supportive of OP.

## 1. Introduction

Outdoors active play (OP) and independent mobility are important aspects of children’s development. In addition to children being more physically active [1,2,3,4], which directly impacts on their physical health [5], OP exposes children to informal social engagement without adults intervening, provides opportunities to resolve conflicts, opportunities for creativity, [6,7] and increases children’s self-confidence and road safety skills [8]. From a public health perspective, increasing OP and active travel by children and youth has been suggested as a strategy to combat the epidemic of childhood obesity [4,9,10,11]. Enhancing children’s OP is particularly important given the alarming declines in children’s walking and cycling to destinations [8,12] and generational declines in OP in favor of sedentary screen-based play [13].

In order to avert these trends, it is important to understand the influences of these behaviors. While numerous reasons at the child′s and family level may explain these changes [9], in recent years a greater attention has been given to modifiable environmental influences on physical activity and active mobility, which has been summarized by a number of reviews [14,15,16]. Some inconsistencies between study findings were noted, a phenomenon that was attributed to the variability in measurement methodologies as different studies use subjective or objective measures of the environment and physical activity behaviors [14]. However, across reviews consistent relationships were noted between neighborhoods’ supportive facilities (e.g., walking and bike paths, distance to destinations and traffic speed/road safety) with children’s active travel [15,16] or physical activity in general [14]. The review by Ding et al. (2011) also highlights that objectively measured land-used mix and neighborhood density are consistently positively associated with physical activity among children (3–12) and youth (13–18) [14].

Informal OP is one mode of the leisure-time physical activity and was assessed in several studies included in this review [14]; however, the specific environmental influences of this form of activity were not examined separately. This is important given the theory and findings that environmental influences on physical activity are domain and context-specific [17,18]. In this respect, it was hypothesized that children’s OP will be higher in suburban neighborhoods characterized by cul-de-sac streets, low traffic volume and greater exposure to open green space. On the other hand, such suburban environments may inhibit children′s independent mobility, due to low density, predominantly residential land use and poor access to destinations. These competing influences of suburban environments may present challenges to urban planners [19].

Only few studies to date were set up to examine this assumption. A literature review that compared different built environments [20] found that relative to urban children, suburban children demonstrate higher rates of physical activity and rural children show higher rates of OP. These findings have been attributed to parent’s perceptions of safety, which were better in suburban neighborhoods compared to inner city neighborhoods [21].

Several environmental factors were found related to children’s OP. Overall, studies among children aged 6 to 12 years old in the US [19], Canada [22,23], UK [24] and Australia [25] consistently suggest that informal OP is more common in suburban neighborhoods, characterized by low-density and cul-de-sac street patterns, compared to traditional neighborhoods, characterized by high-density and grid street networks. In the Netherlands, OP was dependent on the presence of sidewalks and traffic safety [26] or the diversity of routes [27], green open spaces and play areas [28]. In the US Midwestern area two retrospective studies of college age students inquiring about their childhood experience with OP found large variations in the frequency of OP between residential settings. For example, playing with snow, or climbing trees were more frequently experienced by students who lived in rural areas, following by those living in suburban or small town areas and those living in large cities reported the least frequent OP [29].

The above studies however relied only on parents’ perceptions of the environment. This may undermine other environmental influences on children who had already gained some independent mobility (approximately between the ages of 9–12) and could choose for themselves where, when and how much to play. For example, a focus group study among children aged 10–11 years old in Bristol, UK revealed that fear from groups of teenagers may constrain children’s outdoor active play. Additional findings of this study support the importance of green space and cul-de-sac streets as promoters of outdoors active play (Brockman, Jago and Fox, 2011). In Auckland, New Zealand a comprehensive approach to uncover the influences of independent mobility and OP was undertaken in the “Kids in the City” project [30], which included mapping of neighborhood physical characteristics using Geographical Information System (GIS), children’s route diaries and small group interviews of children’s experience in both suburban and inner-city neighborhoods. The findings revealed that a variety of destinations were all important places to play and socialize, as long as they feel safe from intimidating strangers. Such destinations included green open spaces as well as: malls, community churches, local stores, streets, driveways and building corridors. However, there were striking differences by neighborhood socioeconomic status (SES) with regard to children′s independence and OP, as children from low SES were more likely to move independently in their neighborhoods and play outdoors than children in higher SES neighborhoods [31].

Following the example from Auckland, this study also used multiple methodologies with the focus on children rather than parents. The main aim of this study was to explore environmental and psychosocial attributes related to OP, while comparing between inner-city and suburban neighborhoods. Specific aims were:

1. To describe children’s OP habits, while distinguishing between different locations and comparing between inner-city and suburban neighborhoods. Based on previous studies [19,24,25], it was hypothesized that OP will be more common in suburban neighborhoods.
2. To identify psychosocial and sociodemographic factors associated with OP. We hypothesized, based on preceding research, that OP will be more common among boys than girls [1,32,33] and will be associated with independent mobility [1] and with children’s perceptions of the environment [3].
3. To map specific places that were reported by children as play areas (henceforth: reported play areas) in both neighborhood types. We hypothesized that suburban children will have more places where they regularly play outdoors compared to inner-city children. This hypothesis was based on a previous analysis of the study area [34,35], showing that the suburban neighborhood provides more supportive infrastructure for OP, such as multiple green open spaces and cul-de-sac streets.
4. To explore children’s experience of OP in suburban and inner-city neighborhoods, while describing barriers, facilitators as well as other aspects of OP. This part of the study was exploratory and descriptive by nature, and thus, no specific hypotheses were predetermined.

## 2. Materials and Methods

### 2.1. Study Area and GIS Data

The study was conducted in the city of Rishon LeZion, the fourth largest city in Israel (228,200 inhabitants), located along the central Israeli Coastline plain, 12 km south of Tel Aviv. The study area consisted of seven neighborhoods, including four “inner-city neighborhoods”, characterized by high density, land-use mix and grid street network, and three “suburban neighborhoods”, characterized by low density, land-use segregation, and cul-de-sac streets. The neighborhoods were chosen so as to have similar socio-economic indicators, including the percent of participants in labor force (97.3–98.4% in traditional neighborhoods, and 97.4–98.8% in suburban neighborhoods), and the percent of recipients of an undergraduate academic degree (27.8–31.4% in the traditional neighborhoods, and 23.4–30.7% in the suburban neighborhoods). Additionally, these neighborhoods do not have public housing on their premises.

GIS analysis was used to describe the urban form and use measures at the neighborhood level, as presented in Table 1.

Urban form measures included residential density (number of households per sq km), built coverage (overall built area per built lots area) and street connectivity (number of intersections per sq km). Land use measures included the percent of land use dedicated for: retail, green open space and public facility. GIS was also used later in this study as the mapping activity findings were coded into GIS for presentation purposes. GIS data was provided by the city of Rishon LeZion Municipality and included urban form indicators on the one hand, and land use measures on the other. 

As presented in Table 1, the study area included 7 neighborhoods—4 inner-city and 3 suburban neighborhoods that participated in the first phase of the study (school survey). Two out of the seven neighborhoods, including 1 inner-city and 1 suburban neighborhood, also participated in the second phase of the study (semi-structured interviews and mapping activity).

### 2.2. Study Design and Procedure

This mixed method study used a sequential explanatory design [36], in which quantitative data collection and analysis was followed by qualitative methods in an attempt to explain the quantitative results. Specifically, data was collected in two phases; first, a comprehensive quantitative pen and paper survey was held followed by a mapping activity and complementary semi-structured interviews. The survey took place during September 2010 to January 2011, and included 573 5th (10–11 years old) and 6th graders (11–12 years old) from 4 inner-city (*n* = 283) and 3 suburban neighborhoods (*n* = 290) (one school within each neighborhood). Semi-structured interviews (and mapping activity) were conducted during May to June 2011 among an independent subsample of the survey sample, including 5th and 6th graders (*n* = 80) from two schools located in two out of the seven neighborhoods in the study area—one inner-city (*n* = 40) and one suburban (*n* = 40).

#### 2.2.1. School Survey

Within each neighborhood, one primary school was chosen to participate in the survey (overall 7 schools). Four classes per school participated in the survey, including 2 classes of 5th grade and 2 of 6th grade. Prior to the survey, school principals and teachers were provided with information regarding the study, and two weeks before the survey a passive consent letter was sent to parents. Ethics approval was received from the Technion Ethics Committee and from the Israeli Ministry of Education.

Self-administered questionnaires were completed in classrooms under exam-like conditions. For each class, three investigators plus the classroom teacher were in attendance to give assistance to the children when required. In order to ensure that the questions were understood and answered accurately as intended, the first author (MM) gave instructions and read the questionnaire out loud, while two research assistants provided students with individual help when needed. Each class filled out the survey during one school hour (a 50-min period).

##### Survey Measures

The survey included questions regarding OP and several psychosocial and sociodemographic variables that are known to be related to children’s OP. The survey questionnaire was developed based on existing literature and pilot tested among 60 pupils (30 5th graders and 30 6th graders) from two out of the seven schools participating in the study (one school located in a traditional neighborhood and the other school located in a suburban neighborhood).

*Location based outdoors play*: Children were asked how often they play outdoors in the afternoon in three different types of locations, including: (1) recreational facilities (i.e., park, playground, courtyard); (2) public facilities (i.e., community center, library, culture center); and (3) streets within the home neighborhoods. For each of the three locations, children were able to select one of the four options: “0 = never”, “1 = once in two weeks at most”, “2 = 1–2 times a week”, and “3 = at least 3 times a week”.

*Psychosocial and sociodemographic factors*: the survey included items on perceptions of the neighborhood environment independent mobility.

*Perceptions of the neighborhood environment as child-friendly*: assessed through five statements to which the respondents were asked to agree or disagree, as follows: (1) “There are many places that I can walk to in my neighborhood”; (2) “There are many interesting things to look at while walking in my neighborhood”; (3) “There are many parks and playgrounds in my neighborhood”; (4) “There are many places in my neighborhood where it′s nice to play outside in the afternoon”; (5) “It’s fun to play out in the streets of my neighborhood”. These statements referred to the neighborhood environment defined as an area within a 10 min walk around the respondents’ home. The five statements yielded fair intra-class reliability (Cronbach’s Alpha = 0.6, *N* = 573). Each of these statements was coded as 0 or 1 (disagree or agree, respectively), and they were all added up, yielding a composite measure of “perceptions of the environment as child-friendly” with values ranging from 0 to 5, where 0 = “the neighborhood environment is perceived as not at all child-friendly”, and 5 = “the neighborhood environment is perceived as very child-friendly”. This measure was then categorized dichotomously by using the median value (4) as a cut-off.

*Independent mobility*: defined as the extent to which the child is allowed by his/her parents to walk or bike alone in the neighborhood environment [37]. Independent mobility was assessed by one dichotomous variable based on the respondents′ agreement with the following statement: “I am regularly allowed to walk alone during daytime” (0 = disagree, 1 = agree).

Finally, the survey contained a few questions regarding sociodemographic characteristics, including gender, grade and number of cars per household.

#### 2.2.2. Mapping Activity and Semi-Structured Interviews

This part of the study was conducted among an independent subsample of the survey sample, including 80 children from two neighborhoods—Abrabmovitch (*n* = 40) and Neot Shikma (*n* = 40) (see Table 1). These two neighborhoods were chosen given their different built environments (as presented in Table 1) along with the similar characteristics of their residents, which enables exploring environmental influences on human behavior while controlling for social aspects. The mapping activity and semi-structured interviews took place during school hours and were facilitated by the first author (Mika R. Moran). Prior to data collection, school principals and teachers were provided with information regarding the study, and consent forms and information regarding the study were delivered to the children’s parents through the school. Children participated in this study only after providing a signed informed consent from their parents.

This mapping activity lasted about 10 min and took place during school hours in small groups of up to 7 children. A list of children who provided parental consent was used to randomly select participants for the mapping activity and semi-structured interviews. During this activity, participants were provided with a street map of their neighborhood upon which they were asked to mark places where they regularly play and/or hang out with friends (henceforth: “reported play areas”). Although the mapping activity was conducted in small groups, each participant completed the procedure individually. In order to avoid interactions and mutual influences among participants, it was clearly stated that this was an individual activity that each participant needs to complete on his/her own. Following the mapping activity, selected participants were invited to brief one-on-one semi-structured interviews to further explore how they experienced their reported play areas and OP in general. During the interviews, the interviewer (MM) wrote down all of the things that the participants said.

### 2.3. Analysis Plan

Correspondingly to the data collection, data processing and analysis was mixed and included quantitative and qualitative methodologies, as well as GIS coding and analyses.

Statistical analysis of the survey findings was conducted by using SPSS 20.0. (IBM Corporation, Armonk, NY, USA Descriptive statistics were obtained for OP and chi-square tests were used to determine the bi-variate associations between OP with neighborhood type, psychosocial and sociodemographic variables (independent mobility, perceived environment, and number of cars per household). Three multivariate logistic regression models were applied to examine the independent contribution of each variable to playing outdoors at least three times a week as opposed to less frequent play. In the bivariate analysis, OP (in the different locations) was defined according to the respondents’ answers in the survey as a categorical variable with four values. In the multivariate analysis, OP was defined as regular playing (playing outdoors at least three times a week against all other).

The mapping activity and semi-structured interviews were analyzed in a descriptive and qualitative approach. The play areas reported by each participant were coded into GIS to create a point layer reflecting the quantity, variety and spatial distribution of reported play areas (aim 3). In addition to physical locations (X and Y coordination), characteristics of play areas were documented in the layer’s attribute table, including the type of setting (e.g., park, basketball court, plaza) the number of participants who mentioned the play area, etc. Content analysis of the semi-structured interviews was employed to explore the experience of OP in general and in selected reported play areas in particular (study aim 4). The content analysis was performed in an inductive approach by moving from the specific transcripts to more general themes. First, specific elements related to OP were identified in the transcripts and classified as either facilitators, barriers or other. These specific elements were then grouped based on similarity to create six sub-themes, which were later grouped to form three Themes. To ensure inter-rater reliability, the transcripts were read by two independent researchers and themes were identified and verified through discussion and mutual consensus.

## 3. Results

### 3.1. Survey Findings

The findings presented here concern 573 children who participated in the survey and were found eligible to be included in the analysis (see description of inclusion criteria at [35]: Of the 573 children who participated in the survey, 49% (*n =* 283) lived in inner-city neighborhoods, and 51% (*n =* 292) lived in suburban neighborhoods. The sample included similar numbers of boys and girls (*n =* 287, 50% and *n =* 286, 50%, respectively), and 5th and 6th graders (*n* = 294, 51% and *n =* 279, 49%, respectively), which remained similar in both inner-city and suburban neighborhoods. Most of the participants (*n =* 320, 59%) reported having more than two cars in their household, and this percentage was significantly higher in suburban compared to inner-city neighborhoods (68% (*n =* 198) vs. 44% (*n =* 122), *χ*^2^ = 43.32, *p* < 0001).

Table 2 presents descriptive statistics of OP as reported in the school survey, while providing chi-square comparisons of OP by neighborhood type, psychosocial and sociodemographic variables. Overall, OP was found to be uncommon among the survey sample. The most common location for OP was recreational facility (i.e., park, playground), with 40% of the total sample reported playing there at least 3 times a week, followed by “public facility” (i.e., school yard, community center plaza)—reported by 26% of the total sample; and streets—reported by 17% of the total sample.

Surprisingly, no differences were observed between the two neighborhood types in OP. As shown in Table 2, “never playing outdoors” was more common in inner-city neighborhoods compared to suburban neighborhoods, especially when it comes to playing in streets (43% vs. 32%) and in public facilities (28% vs. 19%). However, these differences were not significant. 

Psychosocial factors were found to be associated with OP in different locations. As presented in Table 2, OP in all three locations was significantly positively associated with independent mobility and with children′s perceptions of the environment as “child friendly” (i.e., there are many parks and playgrounds, places to play and nice things to look at in the neighborhood). 

Regarding sociodemographic factors, OP was significantly more common among boys in all three locations. Child’s age (as represented by grade) and number of cars per household were not associated with OP. It is noteworthy that independent mobility and perception of the environment as children friendly were significantly higher in suburban neighborhoods. However, no significant interactions were observed between these variables in predicting OP in all three locations (findings not presented).

Table 3 presents the independent contribution of psychosocial and sociodemographic variables in predicting location based OP as manifested by the odds ratio, which represents the ratio of the probability of playing outdoors at least three times a week to that of not doing so. In all three models, OP was significantly associated with all of the variables in the models. Gender was found to be the strongest predictor of OP in a park and in a public facility, and independent mobility was the strongest predictor of OP in streets. The perceived environment was the weakest predictor of OP in all three locations. Given that, as aforementioned, suburban children had higher independent mobility and better perceptions of the environment, we also examined interactions between neighborhood type with independent mobility and perceived environment in predicting OP. However, no significant interactions were found (results not reported).

### 3.2. Findings from the Mapping Activity and Complementary Semi-Structured Interviews

80 children aged 10–12 years old (5th and 6th graders) participated in the mapping activity and complementary semi-structured interviews. Participants were recruited from two primary schools, one of which was located in a suburban neighborhood (Neot Shikma, *n =* 40) and one in an inner-city neighborhood (Abramovitch, *n =* 40). Participants were equally distributed by age and gender within each neighborhood.

#### 3.2.1. Reported Play Areas

**Figure 1:**
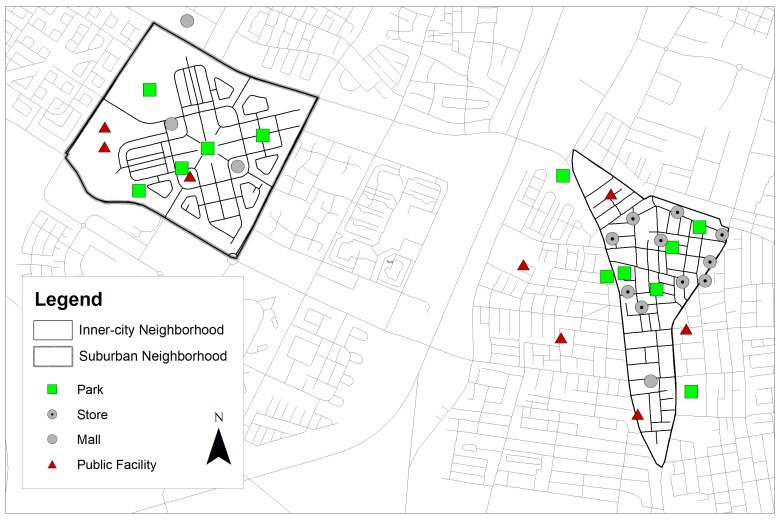
Reported play areas.

Figure 1 presents the play areas reported by participants in the two neighborhoods. Overall, inner-city participants described (more than two times) more OP areas compared to suburban participants (23 vs. 11). Of the 23 play areas reported by inner-city participants, nearly half (*n =* 10) were located in streets near retail shops (henceforth: retail street segment), 7 were located in parks, 5 in public facilities and 1 was in a mall. Of the 11 play areas reported by suburban participants, nearly half (*n =* 5) were located in parks, 3 were located in public facilities and the remaining 3 were located in malls. The types of reported play areas within each neighborhood well correspond with the built environment in those neighborhoods, given the high proportion of retail land use in the inner-city area on the one hand, and the high proportion of green open space in the suburban area on the other hand (Table 1). Having said that, when looking at the actual counts of parks that were reported as play areas, inner-city participants mentioned more parks compared to their suburban friends (7 vs. 5). This, again, stands in contrast to the abundant park area in the suburban neighborhood compared to that in the inner-city neighborhood (32.96% vs. 3.92% of the total neighborhood area, respectively (see Table 1)).

From a more regional perspective, it is noteworthy that the great majority (10 out of 11) of play areas reported by suburban participants were located within their home neighborhood, while inner-city participants described quite a few play areas (5 out of 23) outside of their home neighborhood (Figure 1); these included three parks and two public facilities.

#### 3.2.2. Findings from Complementary Semi-Structured Interviews:

Table 4 presents the themes, subthemes and selected quotes. Content analysis revealed the following three themes and corresponding sub-themes that were described as related to OP: (1) Play areas: Public parks and playgrounds, informal play areas in common areas of residential buildings; (2) Other people: Presence/absence of other people, presence of intimidating groups in parks, presence of parents/grandparents with young children in parks; and (3) Social norms: Low social acceptability of OP, increase in indoors passive leisure. As shown in Table 4, most of the subthemes were classified as barriers and/or facilitators for OP, while a few themes didn′t qualify as neither barrier nor facilitator, and thus were classified as “other”. It is noteworthy that all of the themes and subthemes were commonly reported by participants from both neighborhood types, except for two subthemes that were reported only by inner-city participants, namely the creation of informal play settings in common areas of residential buildings (Table 4, 1.2) and the presence of parents/grandparents with young children in parks (Table 4, 2.2).

##### Play Areas

Participants described high quality and well-maintained parks and playground as enhancing OP by providing opportunities for various activities (Table 4, quotes 1.1.1 and 1.1.2). On the other hand, lack of play structures within playgrounds was described as a barrier (Table 4, quotes 1.1.3). Interestingly, inner-city participants described a phenomenon in which informal play areas created in common areas of residential buildings, such as building lobbies, entrance halls, or shelters (Table 4, quotes 1.2.1 and 1.2.2).

### 3.3. Presence/Absence of Other People in Public Space

Participants described several mechanisms through which the presence/absence of other people may impact the sense of personal safety. The presence of other people was described as increasing personal safety, as other people may take care of each other and call for help if needed (Table 4, quotes 2.1.1 and 2.1.2). For the same reason, the absence of other people was described as a barrier, as when there′s nobody around, no one can help you if needed (Table 4, quotes 2.1.3).

Two distinct population groups were mentioned as inhibiting OP. First, participants from both neighborhood types mentioned the presence of intimidating groups, such as older children and teenagers that may harass them when playing in parks (Table 4, quotes 2.2.1 and 2.2.2). Interestingly, inner-city participants also described the presence of parents and grandparents with younger children in the park as a barrier, as many times they (participants) were asked to stop playing around too fast so they won’t hurt the little ones (toddlers) (Table 4, quotes 2.3.1 and 2.3.2).

### 3.4. Social Norms

Participants mentioned unsupportive social norms concerning OP. On the one hand, participants described low social-acceptability of OP, as it was perceived by participants (and their parents) as an inferior type of leisure activity that was associating it with “non-normative’ behaviors and "street kids" (Table 4, quotes 3.1.1 and 3.1.2). Furthermore, for these reasons, children mentioned that their parents do not allow them to play outside. On the other hand, participants described a shift from active outdoors to passive indoors leisure. This was mainly reported by 6th graders from both neighborhood types, explaining that they don’t play outdoors as much as they used to, and instead they hang out with friends at their home or go out together to buy ice-cream or to see a movie (Table 4, quotes 3.2.1 and 3.2.2).

## 4. Discussion

This study did not find that frequent OP is more prevalent in suburban environments despite the greater exposure to green space and the (assumed to be safer) cul-de-sac streets in the selected suburban communities. Instead, we found that being a boy, having greater independent mobility and the perception of a child-friendly environment were strongly associated with frequent OP. Interviews and mapping activities complemented these quantitative findings by showing that abundant green space alone is not sufficient if there are no people around or if intimidating groups are present, as well as the existence of norms that view OP as unwarranted behavior.

Unexpectedly, and in contrast to our first hypothesis, the survey showed that OP was not more common in suburban neighborhoods compared to inner-city neighborhoods. These findings are opposed to previous studies [19,24,25] and are surprising given the supportive infrastructure in suburban neighborhoods (Table 1) along with the survey findings (not reported here) indicating that suburban participants had higher independent mobility and perceived their neighborhood environment as more child friendly. These surprising findings may be explained, at least partially, by the findings of the mapping activity that show that suburban participants reported having much fewer reported play areas than their friends who live in the inner-city neighborhood (11 vs. 23). From here it may be hypothesized that the suburban children do not spend more time playing outdoors because they don’t have many options or varied options where they can play. This hypothesis may be tested in future research by comparing the frequency of OP across different combinations of neighborhood type by number of reported play area (e.g., inner and low play areas, suburban and low play areas, etc.).

**Figure 2:**
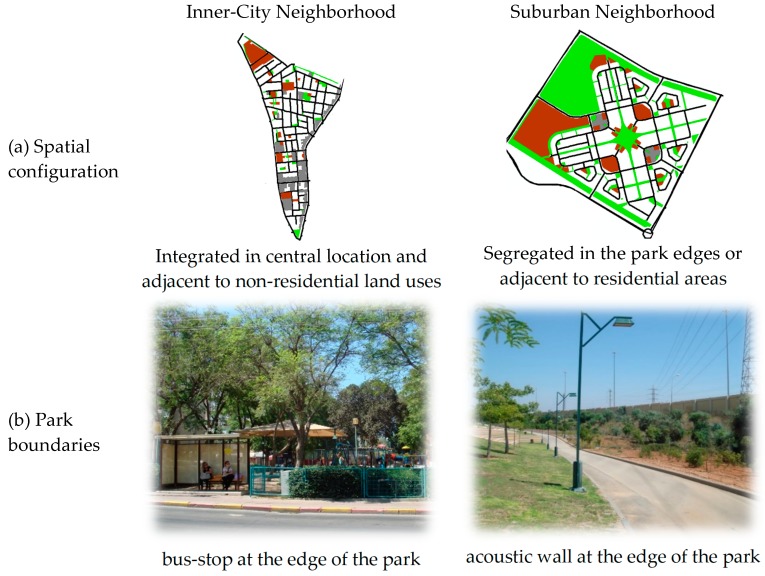
Spatial configuration and internal characteristics of parks in suburban and inner-city parks.

In spite of the relatively high amount of green open space in suburban compared to inner-city neighborhoods, suburban children did not play outdoors more often at parks and reported a smaller number of parks that they regularly play at. This dissonance may be attributed to the spatial configuration and characteristics of parks in the two neighborhood types (Figure 2).

Regarding their spatial configuration (Figure 2, row a), suburban parks are located in segregated areas either at the edge of the neighborhood or adjacent to residential areas, while inner-city parks are integrated in the urban fabric in central locations or adjacent to shops, public facilities and other non-residential land uses. Regarding the parks′ characteristics (Figure 2, row b), suburban parks often have clear boundaries, such as acoustic walls or roads, which isolate it from its surroundings and provide barriers for pedestrians. Inner-city parks, on the other hand, have open and interactive boundaries consisting of local streets with pedestrian oriented design, including bus stops, shops, etc. These differences in parks’ spatial configuration and characteristics may result in decreased and enhanced pedestrian activity in suburban and inner-city parks, respectively, and thereby influence children’s use of parks. Indeed, participants in the semi-structured interviews described the absence of people in public space as decreasing the sense of personal safety. Therefore, it may be assumed that these factors interact in a negative feedback process as follows: First, the spatial segregation and isolation of suburban parks makes them less appealing for pedestrians. The lack of pedestrians in suburban parks then decreases the chances that other pedestrians will visit those parks, given that walkability studies support the notion that seeing more people walking increases the likelihood of a person to walk [38]. At the same time, the relatively “empty” parks may attract less normative populations (such as gangs), which add to the low sense of personal safety. A similar, but opposite, process may occur in inner-city parks: the spatial integration of these parks attracts pedestrians and human activity, thereby creating a high sense of personal safety, which further attracts more “normative” pedestrians and so on and so forth. Although this feedback process is hypothetical, the current study along with previous studies provides evidence to partially support it [39,40]. For example, Rofe et al. examined perceptions and use of green open spaces in Israeli cities, and found that green public open spaces were most intensively used by pedestrians when these were combined with civic/paved public open spaces (e.g., civic squares, market places, promenades and pedestrian streets).

**Figure 3:**
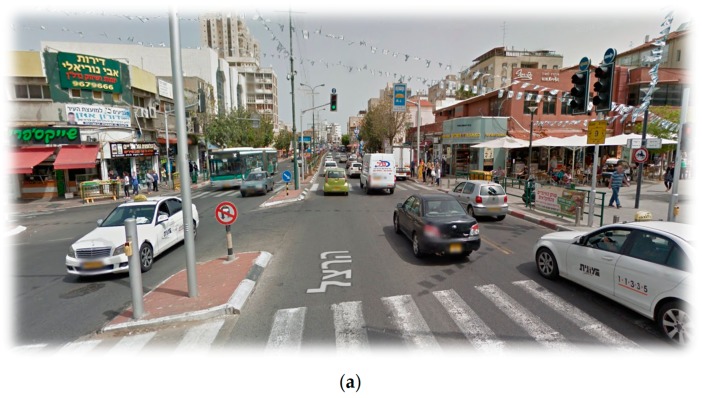

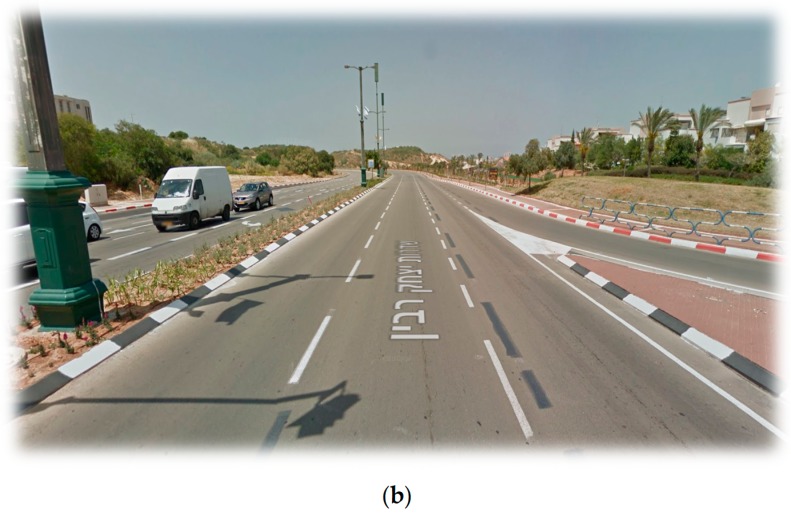
Neighborhood boundaries: (**a**) Inner-city neighborhood boundary with pedestrian-oriented design; (**b**) Suburban neighborhood boundary with automobile-oriented design. (Source: Screen captures of Google Street View panoramic images).

Interestingly, in the current study, suburban children reported playing in areas within their home neighborhood, while inner-city children described playing also outside of their neighborhood. This may reflect the potential impact the neighborhoods’ boundaries on children’s activity space. As shown in Figure 3, the two neighborhoods differ dramatically in the type of their boundaries: While the inner-city neighborhood consists of a dense urban fabric that is similar to its surroundings and has no salient boundaries, the suburban neighborhood is surrounded by multilane highways that may provide a physical barrier for pedestrians. This is likely to explain why suburban children do not play beyond their neighborhood area, as previous theoretical and empirical literature have pointed at the adverse impact of highways on pedestrian activity and sense of community, e.g., [38,41,42,43]. For example, previous studies have shown that the presence of multilane roads inhibits children’s active travel to school [44,45]. Furthermore, highways were found to act as wedges within the urban fabric that have a consistent negative effect on neighboring, attachment and cohesion [42].

A novel finding in our study is the association with socio-cultural aspects of children’s OP, suggesting that low social-acceptability inhibits OP despite the child-friendly neighborhood. The fact that OP provides opportunity to socialize was documented in all studies involving children’s perceptions [24,31]. There is limited literature on normative aspects of OP to compare with this finding. In the Netherlands, children’s OP was found to be significantly correlated with parents′ perception of social cohesion. By contrast, parents′ satisfaction with social contact did not explain the variation in OP [27]. When children of similar age were the focus of the interview, such as in the UK, the social barriers of OP were fear from teenagers’ groups. Children described OP entirely positively; “enjoyable”, “sense of freedom”, “break the boredom” and no mentioning of “out of the age norms” [24]. Why OP was a normative concern in Israel is hard to explain, but could be an issue associated with a specific ethnic group.

This study has several strengths and limitations. A major strength of this study lies in its design (i.e., controlling for SES and thereby eliminating a major confounder in environment–behavior research) and the qualitative and quantitative approach combined with objective measures of neighborhood characteristics. While the survey included questions concerning OP and psychosocial and sociodemographic variables, the mapping activity identified the actual locations of OP, and the semi-structured interviews provided complementary information on participant’s perceptions as contextualized in space. Correspondingly, the analysis of these three data-sets (survey, mapping activity, and complementary semi-structured interviews) presents a comprehensive in depth perspective on children’s OP while providing neighborhood differences. Among the limitations is the relatively small sample within each neighborhood which may have influenced our statistical power to detect significant neighborhood design effects on top of demographic and personal level determinants. Second, we were limited to two separate analyses of two independent samples rather than using a qualitative study among a sub-sample of the survey, which could have enabled a direct link between survey and mapping activity findings. This analysis was due to the fact that only some of the children who participated in the survey provided parental consent for the mapping activity and semi-structured interviews. Following this, the mapping and interview activities generated from two neighborhoods may not be generalized to the whole area; it is possible that a random sample of children from all seven neighborhoods will elicit different themes.

## 5. Conclusions

In conclusion, our findings suggest that in order to encourage routine outdoors play, having abundant green open space in the neighborhood is not enough, but rather the open space should be integrated throughout the neighborhoods in central locations and adjacent to other non-residential land-uses (e.g., retail, public facilities). In addition, unsupportive social norms may inhibit outdoor play. Therefore, environmental change to create safe and attractive outdoors play settings should be accompanied by community interventions to enhance social norms that are supportive of outdoors play.

## Figures and Tables

**Table 1:** Neighborhood-level descriptive statistics of Geographical Information System (GIS)-based environmental variables.

		Land Area (sq Km) ^a^	Population ᵇ	Urban Form			Land Uses (Sq Km, Percent)		
				Intersections Density ^c^	Residential Density ^d^	Built Coverage ^e^	Green Open Space	Public Facilities	Retail
**Inner city neighborhoods**	Abramovitch *	0.73	15,532	134.23	17.40	0.44	0.02 (3.92%)	0.07 (13.72%)	0.08 (15.68%)
	**Katzanelson**	0.843	9805	97.25	9.67	0.38	0.06 (9.68%)	0.08 (12.90%)	0.03 (4.84%)
	**Rambam**	1.353	32,546	112.29	17.61	0.37	0.08 (8.17%)	0.13 (13.19%)	0.09 (8.85%)
	**Remez**	1.555	21,203	63.00	13.44	0.37	0.09 (7.68%)	0.17 (13.68%)	0.03 (2.71%)
**Suburban neighborhoods**	**Chataney Pras Nobel**	1.473	5692	59.06	4.73	0.32	0.15 (13.63%)	0.05 (4.54%)	0.003 (0.27%)
	**Neot Ashalim**	0.545	8987	33.06	11.98	0.22	0.07 (15.41%)	0.10 (22.29%)	0.004 (0.91%)
	Neot Shikma *	1.262	14,297	48.33	9.80	0.25	0.32 (32.96%)	0.16 (16.48%)	0.01 (1.45%)

* Neighborhoods that were included in both survey and mapping activity; ^a^ Source: ICBS (Israel Central Bureau of Statistics), 2011; ^b^ Source: ICBS, 2008; ^c^ number of intersections per sq km; ^d^ number of households per residential dunam; ^e^ the proportion of building area from all built lots.

**Table 2:** Frequencies of outdoors play (OP) at different locations, by the research variables.

**Total Sample**		**Outdoors Play at -**											
		**Green Open Space**				**Public Facility**				**Street**			
		**0**	**1**	**2**	**3**	**0**	**1**	**2**	**3**	**0**	**1**	**2**	**3**
		6% (*n =* 35)	24% (*n =* 136)	30% (*n =* 171)	40% (*n =* 227)	23% (*n =* 134)	25% (*n =* 142)	26% (*n =* 146)	26% (*n =* 146)	37% (*n* = 209)	28% (*n* = 156)	18% (*n* = 98)	17% (*n =* 97)
**N’ type**	**Inner-city**	8% (*n =* 23)	25% (*n =* 71)	28% (*n =* 78)	39% (*n =* 108)	28% (*n =* 79)	22% (*n =* 63)	25% (*n =* 71)	25% (*n =* 69)	43% (*n* = 117)	26% (*n =* 71)	15% (*n =* 42)	16% (*n =* 45)
	**Suburban**	4% (*n =* 12)	23% (*n =* 65)	32% (*n =* 93)	41% (*n =* 119)	19% (*n =* 55)	28% (*n =* 79)	26% (*n =* 75)	27% (*n =* 77)	32% (*n =* 92)	30% (*n =* 85)	20% (*n =* 56)	18% (*n =* 52)
**Model summary**		***χ*^2^ = 5.43, *p =* 0.14, *n =* 569**				***χ*² =6.62, *p* = 0.085, *n =* 568**				***χ*^2^ = 6.58, *p =* 0.087, *n =* 564**			
**Gender**	**Boy**	5% (*n =* 15)	15% (*n =* 43)	29% (*n =* 82)	51% (*n =* 147)	20% (*n =* 58)	21% (*n =* 59)	27% (*n =* 77)	32% (*n =* 90)	32% (*n =* 90)	27% (*n =* 75)	20% (*n =* 55)	21% (*n =* 60)
	**Girl**	7% (*n =* 20)	33% (*n =* 93)	32% (*n =* 89)	28% (*n =* 80)	27% (*n =* 76)	29% (*n =* 83)	24% (*n =* 69)	20% (*n =* 56)	43% (*n =* 119)	29% (*n =* 81)	15% (*n =* 43)	13% (*n =* 37)
**Model summary**		***χ*^2^ = 39.12, *p* < 0.0001, *n =* 569**				***χ*^2^ = 14.83, *p =* 0.002, *n =* 568**				***χ*^2^ = 11.18, *p =* 0.01, *n =* 560**			
**IM**	**High**	3% (*n =* 12)	21% (*n =* 74)	31% (*n =* 107)	45% (*n =* 159)	18% (*n =* 63)	26% (*n =* 91)	28% (*n =* 97)	28% (*n =* 98)	28% (*n =* 97)	28% (*n =* 96)	24% (*n =* 83)	20% (*n =* 69)
	**Low**	10% (*n =* 23)	29% (*n =* 61)	30% (*n =* 63)	31% (*n =* 65)	33% (*n =* 70)	23% (*n =* 49)	22% (*n* = 48)	22% (*n =* 47)	52% (*n =* 110)	28% (*n =* 59)	7% (*n =* 14)	13% (*n =* 27)
**Model summary**		***χ*^2^ = 22.16, *p* < 0.0001, *n =* 564**				***χ*^2^ = 16.02, *p* = 0.001, *n =* 563**				***χ*^2^ = 47.05, *p* < 0.0001, *n =* 555**			
**PEChF**	**High**	5% (*n =* 10)	23% (*n =* 46)	25% (*n =* 51)	47% (*n =* 95)	18% (*n =* 36)	25% (*n =* 5)	26% (*n =* 52)	32% (*n =* 64)	29% (*n =* 58)	27% (*n =* 54)	22% (*n =* 43)	22% (*n =* 44)
	**Low**	7% (*n =* 25)	25% (*n =* 87)	33% (*n =* 119)	35% (*n =* 125)	27% (*n =* 95)	25% (*n =* 89)	26% (*n =* 93)	22% (*n =* 79)	41% (*n =* 145)	29% (*n =* 102)	15% (*n =* 52)	15% (*n =* 51)
**Model summary**		***χ*^2^ = 8.51, *p =* 0.037, *n =* 558**				***χ*^2^ = 8.85, *p =* 0.031, *n =* 558**				***χ*^2^ = 12.87, *p =* 0.005, *n =* 549**			

0 = never, 1 = seldom: once in two weeks or less, 2 = sometimes: 1–2 times a week, 3 = often: 3 times a week or more. N’type = neighborhood type; IM = Independent Mobility, PEChF = Perceived Environment (as children-friendly).

**Table 3:** Logistic regressions to predict OP at park, public facility and street (OR (CI)).

	Outdoors Play at Least Three Times a Week at -		
	Park	Public Facility	Street
Gender	2.51 *** (1.70–3.71)	1.78 *** (1.26–2.52)	1.57 * (1.08–2.28)
Independent mobility	1.65 * (1.12–2.42)	1.45 * (1.01–2.07)	2.89 *** (1.90–4.36)
PEChF	1.23 ** (1.06–1.43)	1.17 * (1.01–1.35)	1.29 *** (1.10–1.52)
Model summary	*χ*^2^ = 40.93, *p* < 0.0001, *n* = 554	*χ*^2^ = 22.78, *p* < 0.0001, *n* = 554	*χ*^2^ = 49.12, *p* < 0.0001, *n* = 545

* *p* < 0.05, ** *p* < 0.01, *** *p* < 0.001.

**Table 4:** Findings from semi-structured interviews—themes, subthemes and selected quotes.

Themes	Subthemes	Selected Quotes
1. Play areas	1.1. Public parks and play grounds	1.1.1. Facilitator: *“That park is spacy and nice and there′s lots of things to do there—work out, swing, slide, and there′s also a labyrinth where you can have some time-out alone and enjoy its beauty or you can play games with friends”* (girl, 6th grade, inner-city neighborhood)
		1.1.2. Facilitator: *“That park is fun to hang out in, it′s spacy and there′s lots of things to do there—swing, work out, play ball, or just sit on the grass and chat with friends”* (girl, 6th grade, suburban neighborhood)
		1.1.3. *Barrier: “It’s boring in that park, there’s nothing to do there—only 1–2 swings, and some fitness facilities and that’s it I would have wanted to have more play facilities there”* (boy, 5th grade, inner-city neighborhood)
	1.2. The creation of informal play settings in common areas of residential buildings	1.2.1. Other: *“The tenants in our building turned the buildings shelter into some kind of an indoor playground—they brought old things that they didn′t use anymore (beds, selves, sofas) and when it′s cold outside we play there, and when it′s hot we play in the building′s lobby/entrance hall this way only we (the buildings tenants) can play there because we have the code to enter the building, and other children that we don′t know can′t come in”* (girl, 6th grade, inner-city neighborhood)
		1.2.2. Other: *“We have a huge storeroom that we′ve built in our house, where we keep all kinds of big toys (for example, ride on toys) and all of the children of our neighbors come and play with the toys sometimes, when its rainy we met at the storeroom and play tabletop games, and if it′s not rainy we play at the buildings outdoor entrance hall in our building area we can play freely, and nobody tells us what to do, unlike at the park where parents of little children won′t let us play because they are afraid that we′ll hurt their little children. So the park turned into a place for little children, and I have more fun playing in my building than at the park”* (girl, 6th grade, inner city neighborhood)
2. Other people	2.1. Presence/absence of other people (general)	2.1.1. Facilitator: *"That park is safe because there′s always a lot of people out there so if something happens to you, they can give you help”* (girl, 5th grade, traditional neighborhood)
		2.1.2. Facilitator: *“It′s safe there because there are always people around, so if you fall people can help you there are always people there because there′s a preschool in the area so the teachers can also help you and if, for example, dangerous people come there—they can′t harm you because there are other people in the area that can call the police”* (girl, 6th grade, suburban neighborhood)
		2.1.3. Barrier: *“Usually there are not many people in the streets where I live, so if someone attacks you there won′t be anybody to call for help”* (girl, 6th grade, inner-city neighborhood)
	2.2. Presence of intimidating groups (older kids) in parks	2.2.1. Barrier: *“Many things happened to kids from my class there (in a recreational facility) for example, some boys from my class went to play there in the afternoon, they put their cellular phones aside, and some older kids came, grabbed their phones and harassed them, but they (the boys from my class) managed to run away, but still, their cellular phones were stolen”* (girl, 5th grade, suburban neighborhood)
		2.2.2. Barrier: *“I′m not allowed to walk in that park in the dark it′s either empty, or you have big kids who harass you one time I walked there in the dark and big kids threw detonators at me”* (boy, 6th grade, suburban neighborhood)
	2.3. Presence of parents/grandparents with young children in parks	2.3.1. Barrier: *“That park is boring and not fun and there are a lot of old people there It bothers me when older people are in the park because they don′t let us have fun—they come with their little children and babies and they take them down the slides, and one time they called the police because we were playing ball and they thought that we didn′t let their children go down the slides”* (boy, 5th grade, inner-city neighborhood)
		2.3.2. Barrier: *“In that park the parents or the grandparents of the little children tell us: ′there are little children here, you′re not allowed to play ball, you’re not allowed to be here′, so it turns into a place for small children because we’re not allowed to play there”* (boy, 6th grade, inner-city neighborhood)
3. Social norms	3.1. Low social acceptability of OP	3.1.1. Barrier: *“I′m not allowed to hang out in the streets without notifying my parents about it because my mom tells me that I′m not a* *‘street kid′ I′m allowed to play outdoors for something like two hours, but not for all day long* *”* (girl, 5th grade, inner-city neighborhood)
		3.1.2. Barrier: *“There are older kids there that play with fire and do things that are not for our age they are ′street kids′ I don′t like it there there are always kids that curse and harass other kids, one time a boy threw a water balloon with mud at me”* (girl, 5th grade, suburban neighborhood)
	3.2. Shift from active outdoors to passive indoors leisure	3.2.1. Other: *“We don′t play outdoors anymore, we go out together—for bowling, the movies, or just go out to get ice-cream—we do that instead of meeting at the park to play ball”* (girl, 6th grade, suburban neighborhood)
		3.2.2. Other: *“I stopped going to that park because the kids in my class don’t play outdoors anymore, instead, they go visit other kids in their home or go to the movies together”* (girl, 6th grade, inner-city neighborhood)
